# BRAF Targeting Across Solid Tumors: Molecular Aspects and Clinical Applications

**DOI:** 10.3390/ijms26083757

**Published:** 2025-04-16

**Authors:** Hiba Mechahougui, James Gutmans, Roumaïssa Gouasmi, Laure Smekens, Alex Friedlaender

**Affiliations:** 1 Oncology Department, Geneva University Hospital (HUG), 1205 Geneva, Switzerland; sam.gutmans@gmail.com (J.G.); laure.smekens@hug.ch (L.S.); 2 Cancer Research Center of Lyon, CNRS UMR5286, Inserm U1052, University of Lyon, 69100 Lyon, France; roumaissa.gouasmi@lyon.unicancer.fr; 3 Clinique Générale Beaulieu, 1206 Geneva, Switzerland

**Keywords:** targeted therapy, molecular oncology, precision oncology, tissue specificity, BRAF

## Abstract

BRAF mutations are critical drivers in cancers such as melanoma, colorectal cancer, and non-small-cell lung cancer. The most common mutation, *BRAF V600E*, is a key therapeutic target. Targeted treatments with BRAF and MEK inhibitors have significantly improved progression-free and overall survival in melanoma patients. However, in cancers like metastatic colorectal cancer, BRAF mutations are associated with poor outcomes due to aggressive disease behavior and resistance to conventional chemotherapy. Despite progress, resistance to BRAF/MEK inhibitors remains a major challenge, often driven by secondary mutations in the mitogen-activated protein kinase (MAPK) pathway, activation of alternative pathways such as phosphoinositide 3-kinases (PI3Ks)/protein kinase B (AKT), or changes in the tumor microenvironment. These challenges have motivated ongoing research into combining BRAF inhibitors with immunotherapies to enhance and prolong treatment effectiveness. Future research must also account for the role of the cancer’s tissue of origin, as the biological context significantly influences response to targeted therapies, highlighting the need for a deeper understanding of tumor biology, micro-environment, and genetics.

## 1. Introduction

Cancer remains one of the leading causes of morbidity and mortality worldwide [1], driven by a complex interplay of genetic, epigenetic, and environmental factors. Historically, cancer treatment has relied on non-specific modalities such as chemotherapy and radiation, which, while effective in some settings, offer limited precision and are often associated with significant toxicity. Over the past two decades, advances in cancer genomics have reshaped our understanding of tumor biology, paving the way for precision oncology, an approach that seeks to tailor treatment based on the specific molecular alterations driving an individual patient’s tumor. Among the most clinically actionable oncogenic drivers identified to date is BRAF, a serine/threonine kinase that plays a pivotal role in the MAPK signaling pathway. Initially discovered in melanoma, BRAF mutations have since been identified across a wide spectrum of malignancies, including non-small-cell lung cancer (NSCLC), colorectal cancer (CRC), thyroid cancer, gliomas, other rare cancers, and various hematologic and pediatric tumors. The discovery of these mutations has enabled the development of targeted therapies, which have transformed outcomes in selected patient populations. This review provides an overview of the molecular diversity of BRAF alterations, their biological implications across tumor types, and the therapeutic strategies that have emerged in response. From melanoma to rare cancers, we highlight how BRAF has become a paradigm of precision oncology.

## 2. BRAF Biology and Its Therapeutical Implication

Throughout their progression, cancer cells experience extensive genetic and epigenetic alterations. The Cancer Genome Atlas (TCGA) detailed molecular characterization of cancers, aiding in classifying tumors for treatment and predicting response to therapy. A multitude of oncogenes and tumor-suppressor genes have been identified across cancer types; however, the interactions between oncogenes and tumor suppressors, especially with tumor heterogeneity and evolution, present a challenge. While numerous non-synonymous mutations exist in cancer cells, only a subset are driver mutations that are critical for cancer development and progression, as opposed to passenger mutations that accumulate without impacting tumorigenesis. Mutant BRAF is a classic driver oncogene with a high prevalence in melanoma (more than 50%), thyroid cancers (50%), colorectal cancers (15%), and NSCLCs (5–8%) [2,3].

**Figure 1:**
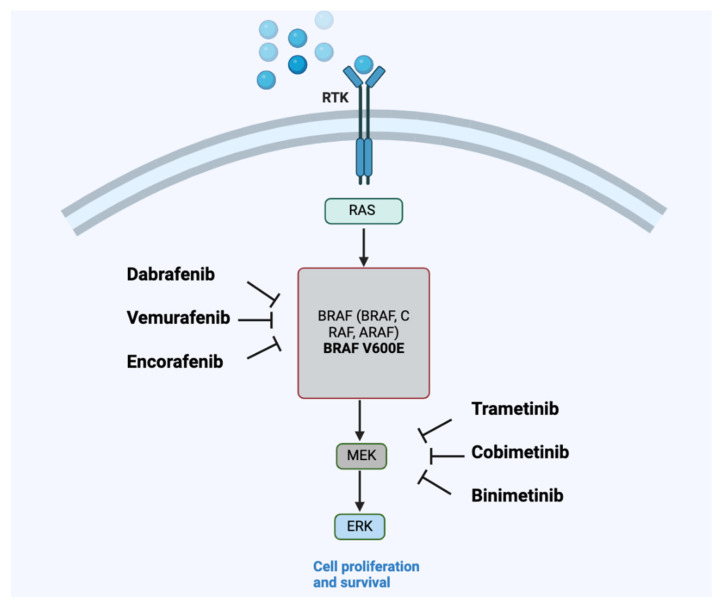
Mechanism of action of anti-BRAF/MEK drugs on the RAS signaling pathway. RTK, receptor tyrosine kinase (including vegFR, PDGFR, and EGFR); RAS, rat sarcoma virus protein; BRAF, v-Raf murine sarcoma viral oncogene homolog B (including BRAF, CRAF, and ARAF); BRAF V600E, a common activating mutation in BRAF; MAPK, mitogen-activated protein kinase; ERK, extracellular signal-regulated kinase. Figure made with BioRender.

In mammals, the RAF protein family comprises three members: ARAF, BRAF, and CRAF (also referred to as RAF1; Figure 1), each sharing three signature domains. These include the CR1 domain responsible for RAS GTP-binding and self-regulation; the CR2 domain, which is a serine-rich hinge region; and the CR3 domain, which is a catalytic domain for serine/threonine phosphorylation of target proteins [4]. BRAF is the predominant RAF kinase that undergoes mutation. Normally, RAF proteins exist as inactive monomers in the cytoplasm, bound to regulators like MEK and heat shock protein (HSP) 90. Upon activation of receptor tyrosine kinases, RAS becomes active and promotes RAF dimerization, converting RAF into its active form. Activated RAF then phosphorylates mitogen-activated extracellular signal-regulated kinase (MEK) 1/2, which in turn activates extracellular signal-regulated kinase (ERK) 1/2, promoting cell proliferation and survival [5,6]. The unique role of BRAF as an oncogene might come from its specific regulation within tissues, its expression patterns, or its distinctive capacity to activate MEK.

In contrast, CRAF and ARAF may have broader functions similar to those of housekeeping genes [7]. The BRAF gene, encoding a protein crucial in the MAPK/ERK signaling pathway, is activated by growth factors interacting with receptor tyrosine kinases, leading to cell growth signaling. The RAS/RAF/MAPK signaling cascade can be activated via two distinct mechanisms: a ligand-dependent route, initiated when ligands such as growth factors, hormones, or cytokines bind to their corresponding cell-surface receptors; and a ligand-independent route, triggered by environmental stressors like radiation or tissue injury [8]. Dysregulated activation of RAF, commonly arising from mutations in RAS or in upstream receptors, particularly receptor tyrosine kinases (RTKs), is critically involved in cancer progression [9]. Key growth factors, including epidermal growth factor (EGF), vascular endothelial growth factor (VEGF) [6], and platelet-derived growth factor (PDGF-β) [10], stimulate this signaling through specific receptors, such as the epidermal growth factor receptor (EGFR), thereby promoting tumor cell proliferation, survival, and metastatic potential [6].

BRAF, as part of the RAF protein family, plays a pivotal role in signaling through its kinase activity [4]. Mutations in BRAF are classified into three distinct classes based on their effects on the MAPK pathway. Class I mutations operate autonomously from upstream RAS signals and can activate the downstream ERK pathway without the need for dimer formation [11]. Due to the negative feedback that activated ERK imposes on RAS proteins, tumors with class I mutations typically have low RAS activity. In such mutations, RAF protein activity is not contingent on an upstream signaling cascade [12]. The most notable example of a class I mutation is V600E, although other substitutions at the same position, such as V600K and V600D, can have similar biological consequences [13,14]. Class II mutations include a broad spectrum of changes, all of which are also independent of upstream RAS signaling. Unlike class I mutations, these require dimerization to initiate activation of the downstream MEK-ERK pathway [15]. Class III mutations, on the other hand, propagate downstream signals by forming dimers with the wild-type CRAF and necessitate upstream activation to heighten ERK signaling. This can occur due to genomic changes, like RAS mutations or NF1 loss, or through RTK signaling, which mitigates the ERK-mediated negative feedback on RAS proteins. Tumors with class III BRAF mutations are typically marked by elevated levels of active RAS-GTP [16,17].

Recently, BRAF inhibitors have received approval based on organ-specific studies [18] and basket trials [19], and even on a tumor-agnostic basis. It remains critical to account for tissue-specific differences in therapeutic response, as such differences help explain the variable efficacy of targeted treatments across tumor types and highlight the need to develop organ-specific combination strategies.

### 2.1. Impact of BRAF Mutation on Prognosis

Before novel systemic treatments significantly improved outcomes in **metastatic melanoma**, a study of 912 patients diagnosed with cutaneous melanoma analyzed the relationships between NRAS and BRAF mutations, tumor characteristics, and survival. Among these patients, 13% had NRAS mutations, 30% had BRAF mutations, and 57% were wild-type. BRAF mutations were more frequent in younger patients, correlated with the superficial spreading melanoma subtype, and exhibited higher mitotic activity. Although melanoma-specific survival did not differ significantly among patients with NRAS, BRAF, or wild-type tumors overall, the presence of NRAS or BRAF mutations in higher-risk tumors (stage T2b or above) was associated with notably increased mortality risk, an effect not seen in lower-risk tumors (stage T2a or below) [20]. Additionally, the two main BRAF mutation subtypes, V600E and V600K, display distinct clinical features; V600E occurs predominantly in younger patients, whereas V600K is common among older patients with chronic sun damage and higher mutational burden, often leading to more aggressive disease but better responses to immunotherapy [21]. BRAF-mutated melanomas carry a worse prognosis and a higher metastatic potential compared to wild-type melanomas [22].

**In advanced colorectal cancer**, BRAF-mutated CRC represents a distinct clinical entity characterized by specific patient demographics, clinical behavior, and prognostic outcomes. These tumors typically occur in older female patients and are commonly located in the proximal colon, often presenting with poor differentiation, mucinous histology, larger primary tumors, and a high frequency of nodal and peritoneal spread [23]. BRAF mutations occur in approximately 8–15% of CRC cases, with the BRAF V600E variant accounting for about 90% of these [23,24]. BRAF-V600E is nearly always mutually exclusive with KRAS [23].

BRAF V600E mutations are significantly more common in microsatellite instability-high (MSI-H) tumors compared to microsatellite-stable (MSS) tumors, due to their involvement in the serrated neoplasia pathway. In this pathway, BRAF mutations initiate serrated adenomas that often develop widespread DNA methylation (CIMP-high), leading to silencing of the MLH1 mismatch repair gene and, consequently, MSI-H status. This indicates a sporadic origin of MSI-H CRC, distinguishing these tumors from Lynch syndrome-related CRC, which rarely carries BRAF mutations [25,26].

Clinically, patients harboring BRAF mutations have significantly lower responsiveness to conventional treatments and markedly shorter survival compared to those without mutations. Specifically, median survival is approximately 13.4 months for patients with BRAF-mutated CRC, compared to around 37 months for those with wild-type RAS and BRAF [27]. Moreover, the 5-year survival rate for BRAF-mutated CRC patients (47.5%) is significantly lower than for patients with wild-type tumors (60.7%) [28].

The realization of particularly poor outcomes in MSS/BRAF-mutated CRC initially emerged from subgroup analyses in clinical trials, consistently indicating notably higher mortality in this subgroup compared to MSI-high or BRAF wild-type CRC [23,29]. Subsequent research identified intrinsic biological factors underlying this observation. MSS/BRAF-mutant tumors possess distinct molecular profiles, often acquiring additional mutations in TP53, loss of the differentiation marker CDX2, increased CK7 expression, and activation of invasive pathways associated with epithelial–mesenchymal transition [30]. While approximately 20% of BRAF-mutant metastatic CRC cases exhibit MSI-H status [10], MSS/BRAF-mutant tumors lack the MSI-driven immune activation and reduced neoantigen load, limiting responsiveness to immunotherapy [31]. Nevertheless, their aggressive clinical behavior is intrinsically driven by these unique molecular alterations, including high metastatic potential and chromosomal instability, beyond their immunotherapy resistance alone [32].

BRAF mutations are rare in **biliary tract cancers (BTCs)**, predominantly found in intrahepatic cholangiocarcinomas (iCCAs) with an overall incidence of about 5% to 7% [33]. In a study of 926 Chinese patients with various hepatobiliary cancers, BRAF-activating mutations were present in 5.5% of the cases. Specifically, the BRAF V600E mutation was found in 1.5% of iCCAs and 0.5% of gallbladder cancers (GBCs), but not in other types of hepatobiliary malignancies [34]. While initial investigations did not find a link between BRAF mutations and survival rates [35], more recent research indicates that BTCs with the BRAF V600E mutation are associated with a more advanced TNM stage, resistance to chemotherapy, a more aggressive disease course, and poorer survival [36]. WT patients had an average survival of 37.3 months, while patients with BRAF-mutated tumors had an average survival of only 13.5 months.

The incidence of BRAF mutations in **non-small-cell lung cancers (NSCLCs)**, particularly adenocarcinomas, has historically been underestimated due to the methods used for mutation detection. Initial studies using RT-PCR targeting exons 11 and 15 alongside High-Resolution Melting Analysis (HRMA) identified BRAF mutations in approximately 5% of lung adenocarcinomas, with about 58% classified as V600E mutations [37]. Subsequent whole-exome sequencing studies suggested a higher prevalence, detecting BRAF mutations in 7–8% of adenocarcinomas, with alterations in the MAPK-ERK pathway present in 76% of these cases [38]. More recently, analysis of circulating tumor cell-free DNA from lung adenocarcinoma patients indicated an even higher prevalence, near 9%; however, only 2% of these were BRAF V600E [16]. In contrast, BRAF mutations are reported far less frequently in squamous-cell lung cancers, possibly due to infrequent molecular testing [39]. Non-V600 BRAF mutations include class II mutations (e.g., G469A/V [40] and K601E [41]) with intermediate or high kinase activity and class III mutations (e.g., D594G and G466V [42]) that are kinase-impaired and RAS-dependent [42]. Patients with non-V600 mutations often have a history of smoking and tend to have more aggressive disease, including a higher rate of brain metastases at diagnosis. BRAF mutations have also been associated with poorer responses to platinum-based chemotherapy, and in 1–2% of cases, BRAF mutations are implicated as a resistance mechanism against EGFR tyrosine kinase inhibitors [18,43,44].

**In thyroid cancers**, BRAF mutation rates vary across thyroid cancer subtypes. A pooled analysis of 29 studies on sporadic adult thyroid cancer reported an overall BRAF mutation prevalence of 44% in papillary thyroid cancer (PTC) and 24% in anaplastic thyroid carcinoma (ATC). BRAF-positive ATCs are thought to arise from pre-existing BRAF-mutated PTCs, as suggested by cases where both tumor components co-exist and share the mutation [45]. Clinically, BRAF V600E has been associated with more aggressive tumor behavior and a worse prognosis [46]. In PTC, it is linked to a nearly threefold increase in disease-specific mortality [47] and is significantly associated with higher rates of recurrence, lymph node metastasis, extrathyroidal extension, and advanced AJCC stage (III/IV) [48]. BRAF V600E mutation also impairs the efficacy of radioactive iodine (RAI) therapy by activating the MAPK pathway, which suppresses expression of the sodium–iodide symporter and thyroid-stimulating hormone receptor, both essential for iodine uptake in differentiated thyroid cells. This silencing of iodine-handling genes is a key driver of RAI-refractory disease [49,50,51].

### 2.2. Implication of BRAF Mutation on Immunotherapy Efficacy

**In mCRC**, the phase II Keynote 164 [52] study explored the effectiveness of pembrolizumab in MSI-H mCRC patients who had previously undergone systemic therapy. Pembrolizumab monotherapy achieved response rates ranging from 20% to 55% in patients with previously treated BRAF-mutant tumors, depending on the number of prior therapy lines, compared to an overall response rate of approximately 33% in the general population. [53]. Similarly, the CheckMate 142 trial assessed the efficacy of nivolumab or ipilimumab/nivolumab in MSI-H pretreated patients. Among patients with BRAF-mutated CRC, the objective response rate (ORR) was 25%. Notably, in the nivolumab/ipilimumab combination group, the ORR for patients with BRAF-mutated tumors reached 70%, outperforming the response in the BRAF wild-type cohort [54]. Furthermore, the phase III Keynote 177 trial demonstrated a notable advantage of pembrolizumab over chemotherapy in first-line treatment for MSI-H mCRC patients, significantly extending the median progression-free survival (PFS) (16.5 vs. 8.2 months), including in the population of patients with the BRAF V600E mutation [55,56]. A meta-analysis tempered these findings, showing no significant difference in ORR between BRAF-mutant MSI-H mCRC and BRAF WT MSI-H mCRC [57]

**In lung cancer**, BRAF V600E typically occurs in never-smokers, with adenocarcinoma histology, and lacks other common driver mutations, resembling other oncogene-driven cancers such as EGFR or anaplastic lymphoma kinase (ALK)-mutated tumors [58]. Although these tumors generally have lower tumor mutation burdens, studies evaluating immunotherapy responses have shown conflicting results. A recent study indicated that BRAF-mutant tumors exhibited similar CD8+ T cells to regulatory T-cell ratios, cytotoxic gene expression signatures, immune suppressive features, and biomarkers associated with immune checkpoint inhibitor response as wild-type NSCLC [59], whereas others report limited efficacy of checkpoint inhibitors in this population [60]. Consequently, clinical practice commonly stratifies treatment based on smoking history: immunotherapy is offered in smokers, while targeted therapy is prioritized for non-smokers with BRAF V600E mutations [61].

**In metastatic melanoma**, the landmark KEYNOTE-006 trial demonstrated that patients, including those harboring BRAF mutations, benefited from immunotherapy. However, this raised an important clinical question regarding the optimal sequence of targeted therapy and immunotherapy in this population [62]. Preclinical in vivo data show that BRAF inhibitors do not impair lymphocyte viability, proliferation, or cytokine production at therapeutic concentrations, nor do they negatively impact serum cytokine levels or leukocyte counts in melanoma patients [63]. Instead, BRAF inhibitors paradoxically activate the MAPK pathway in BRAF wild-type cells, enhancing T-cell activation through increased ERK phosphorylation [64,65,66]. While this would suggest that this paradoxical MAPK activation could favorably modulate the tumor microenvironment and enhance immune checkpoint inhibitor efficacy [66], clinical outcomes have been mixed. Retrospective studies indicated reduced survival when BRAF-inhibitor treatment preceded immunotherapy. Among patients treated sequentially with BRAF inhibitors followed by ipilimumab, median survival was significantly lower in patients unable to complete ipilimumab treatment per protocol (1.2 vs. 12.7 months) [67]. The randomized DREAMseq trial confirmed improved outcomes with upfront immunotherapy versus targeted therapy in advanced melanoma [68]. Furthermore, analysis from the EUMelaReg registry showed higher response rates when immunotherapy was administered prior to targeted therapy [53]. Consequently, current clinical evidence strongly supports initiating treatment with immunotherapy before considering targeted therapy in metastatic BRAF-mutant melanoma.

## 3. Selected Strategies in BRAF-Mutated Solid Cancers

Targeted therapies against BRAF mutations have significantly transformed the clinical management of patients with BRAF-mutant cancers. In certain settings, such as advanced melanoma, these therapies represented a major breakthrough at a time when no highly effective treatment options were available, particularly before the advent of immune checkpoint inhibitors. In other tumor types, BRAF-targeted approaches have introduced new therapeutic possibilities, often with favorable efficacy and distinct toxicity profiles compared to conventional chemotherapies (Table 1).

### 3.1. Melanoma

In melanoma, BRAF mutations are detected in 50–70% of cases [2]. BRAF encodes a serine-threonine kinase located in the cytoplasm. Over 97% of mutations in the BRAF gene occur at codon 600. The predominant mutation, found in approximately 90% of cases, involves a T1799A transversion, leading to the substitution of valine (V) by glutamic acid (E) at position 600 (V600E). Less frequent substitutions include valine to lysine (V600K, ~8–20%), arginine (V600R, ~1%), leucine (V600M, ~0.3%), and aspartic acid (V600D, ~0.1%). Non-V600 mutations are extremely rare [73].

BRAF inhibitors are effective primarily in tumors with V600E mutations because this specific alteration results in a constitutively active monomeric form of the BRAF kinase that signals independently of upstream RAS input. The V600E mutation mimics phosphorylation, locking BRAF in an active conformation that strongly drives the MAPK pathway, promoting uncontrolled proliferation [74]. Because tumors bearing a BRAF V600E mutation are oncogenically addicted to this hyperactivated signaling cascade, it was postulated, and later confirmed, that small-molecule inhibitors targeting BRAF V600E offer an effective therapeutic strategy for these cancers [75].

The Food and Drug Administration (FDA) first approved the BRAF inhibitor vemurafenib in 2011 for the treatment of metastatic melanoma, with dabrafenib receiving approval in 2013 [76,77]. The efficacy of vemurafenib was assessed against dacarbazine, a conventional therapy, in the BRIM-3 trial, a phase III study focusing on patients with metastatic melanoma harboring previously untreated BRAFV600E mutation [78]. The study reported a 6-month OS rate of 84% for the vemurafenib group versus 64% for the dacarbazine group. The 2017 update of the BRIM-3 trial revealed a final OS of 13.6 months for vemurafenib versus 9.7 months for dacarbazine [79], and the impact of vemurafenib on melanoma with brain metastases was confirmed in findings from 2017 [80].

The **BREAK-3 trial**, a phase III study for patients with previously untreated BRAFV600E-mutant unresectable or metastatic melanoma, served as the basis for the FDA approval of **dabrafenib** as a single-agent therapy [70]. This trial showed a median PFS of 6.7 months for dabrafenib, significantly longer than the 2.9 months for dacarbazine. Vemurafenib required more dose reductions but had a similar 3% discontinuation rate. The BREAK-3 trial’s 2013 update reiterated the PFS advantage, with median values of 6.9 months for dabrafenib and 2.7 months for dacarbazine [81]. The **BREAK-MB** phase II trial highlighted **dabrafenib’s** efficacy in treating BRAFV600-mutant melanoma with brain metastases, showing positive outcomes regardless of prior local treatments [82].

**Trametinib** gained FDA approval in 2014 as the inaugural MEK inhibitor for unresectable and metastatic melanoma, following results from the **METRIC** phase III trial. The 2019 update of the METRIC trial showed a median PFS of 4.9 months and a median OS of 15.6 months for trametinib, surpassing the chemotherapy group’s 1.5-month PFS and 11.3-month OS [83]. There was an emergence of acquired resistance and skin toxicities from the paradoxical activation of wild-type BRAF during BRAF-inhibitor monotherapy. One major challenge associated with BRAF inhibitors alone is the development of hyperproliferative cutaneous events, including squamous cell carcinoma and keratoacanthoma. These effects were traced back to BRAF inhibitor-induced paradoxical activation of MAPK signaling in BRAF wild-type cells. In this setting, BRAF-inhibitor treatment promotes RAF dimerization, which results in transactivation of the uninhibited partner and downstream hyperactivation of the MAPK pathway. This aberrant signaling drives the proliferation of keratinocytes and contributes to the development of skin lesions. This led to the exploration of BRAF and MEK inhibitor combinations that helped mitigate this effect by dampening MAPK signaling [74,84]. This approach, which combined **dabrafenib–trametinib** or **vemurafenib–cobimetinib**, received FDA approvals in 2014 and 2015, respectively, for treating unresectable or metastatic melanoma with BRAF mutations.

The **coBRIM** phase III trial permitted the FDA’s approval of vemurafenib–cobimetinib for treatment-naive patients with BRAFV600-mutated metastatic melanoma, showing improved median PFS and OS of 12.3 and 22.3 months, respectively, versus 7.2 and 17.4 months for vemurafenib alone [85,86]. The 2021 updates maintained the efficacy of the **vemurafenib–cobimetinib** combination with notable median OS and PFS benefits [87].

**Figure 2:**
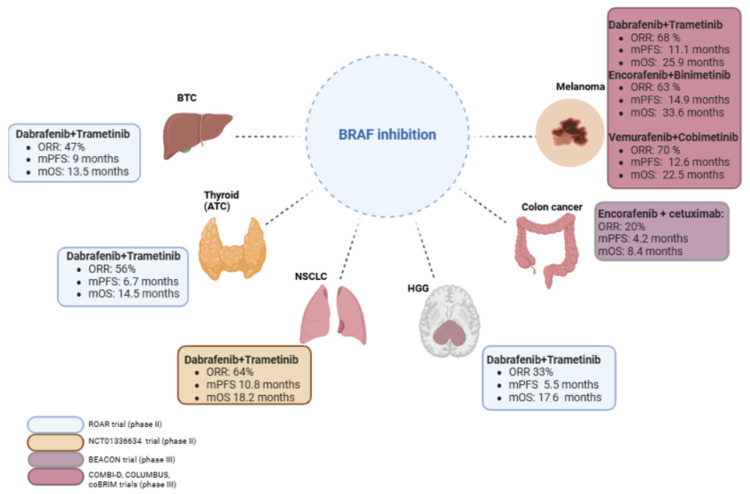
ORR, PFS, and OS in selected trials of BRAF-MEK inhibition in adult solid cancers. BTC, biliary tract cancer; ATC, anaplastic thyroid carcinoma; NSCLC, non-small-cell lung cancer; HGG, high-grade glioma (results for low grade glioma are incomplete). ORR, objective response rate; mPFS, median progression-free survival; mOS, median overall survival. Figure made with BioRender.

The **COMBI-d trial**, a phase III study of patients with BRAFV600-mutated metastatic melanoma, demonstrated that the combination of **dabrafenib and trametinib** had a marginal PFS benefit of 9.3 months versus 8.8 months with dabrafenib alone [88]. The 2017 update validated the combination’s benefit, showing a 3-year PFS of 22% with the combination therapy versus 12% with dabrafenib alone, and a 3-year OS of 44% compared to 32% [89]. The COMBI-v trial showcased superior median OS and PFS for the dabrafenib–trametinib combination compared to vemurafenib alone [90]. The COMBI-MB phase II trial highlighted the efficacy of this combination in metastatic melanoma with brain metastases, though the response duration was relatively brief [91]. The adjuvant potential of dabrafenib-trametinib in completely excised BRAFV600-mutated melanoma was confirmed in the COMBI-AD trial, showing a 3-year recurrence-free survival (RFS) rate of 58% for the combination versus 39% for the placebo, with a 3-year OS of 86% against 77%, leading to its FDA approval in 2018 for adjuvant use [92] (Figure 2).

The FDA approved the encorafenib and binimetinib combination in 2018 for patients with unresectable or metastatic melanoma with BRAFV600 mutations, based on the COLUMBUS phase III trial [93]. This trial compared the combined encorafenib-binimetinib therapy against monotherapies of encorafenib or vemurafenib, revealing a superior median PFS of 14.9 months versus 7.3 months for vemurafenib and 9.6 months for encorafenib alone. The combination showed a 16.7-month OS advantage over vemurafenib, albeit without a significant OS difference when compared to encorafenib alone [94]. The 5-year outcomes from the COLUMBUS trial confirmed the sustained efficacy and durability of the encorafenib and binimetinib combination, with a PFS of 23% and an OS of 35%, in contrast to 10% PFS and 21% OS in the vemurafenib group [95] (Figure 2).

### 3.2. Advanced Non-Small-Cell Lung Cancer

Approximately 5% of patients with NSCLC exhibit BRAF mutations, with BRAFV600E mutations specifically occurring in more than half of these cases [37]. In NSCLC, BRAFV600E mutations tend to be more prevalent in females with a micropapillary histological pattern who do not smoke, whereas non-V600E BRAF mutations are often linked to mucinous histological patterns and are more frequently found in males who have a history of smoking [96].

For V600E mutations in NSCLC, following the successful application of double BRAF/MEK inhibition in melanoma, two phase II trials with dabrafenib and trametinib were conducted on pretreated and treatment-naive patients with metastatic lung adenocarcinoma. The trials showed significant clinical activity; the pretreated group of 57 patients had an ORR of 63% and a 6-month PFS of 65% [18]. The treatment-naive group of 36 patients had an ORR of 64% and a 6-month PFS of 72%. While the impact of BRAF V600E mutations on NSCLC prognosis or chemotherapy efficacy remains unclear, with some studies suggesting shorter PFS with platinum-based chemotherapy [97], this has not been universally supported [98] (Figure 2). Present guidelines in Europe and America recommend first-line treatment with double BRAF/MEK inhibition for NSCLC with V600E mutations [99].

For non-V600E mutations in NSCLC, the prevalent alterations are class II and III BRAF mutants. Despite preclinical studies suggesting that dual MAPK inhibition or monotherapy with BRAF or MEK inhibitors may be effective against class II BRAF mutations in melanoma [100], these approaches are not standard in NSCLC. A case with the BRAF G596V mutation responded partially to vemurafenib [101], and while early studies on BRAF inhibitors in NSCLC were promising [102], only V600E mutations, not non-V600 mutations, showed clinical responses [103]. Another study reported a partial response in V600K-mutated NSCLC and stable disease in V600M and V600D mutations [104]. A sustained response exceeding 15 months was noted in a patient with dual G469A and W604C BRAF mutations treated with double BRAF/MEK inhibition [105]. For class III mutations, which are characterized by elevated RAS activity, combining MEK inhibitors with RTK inhibitors (such as EGFR inhibitors) is considered a potential therapeutic strategy [106].

Sorafenib has been reported to be effective in NSCLC harboring G469R and G469V BRAF mutations in case reports [107,108]. Originally developed as a Raf-1 inhibitor, sorafenib is a multi-kinase inhibitor [109]. This multi-targeted approach allows sorafenib to inhibit both MAPK signaling driven by mutant BRAF and angiogenic pathways critical for tumor growth, offering a mechanistic rationale for its efficacy in BRAF non-V600E-mutant NSCLC [107,108]. An alternative strategy involves targeting downstream MAP kinase pathway signaling, with ERK inhibitors like ulixertinib showing promise in a phase I study, achieving partial responses in patients with both non-V600 and V600E mutations [110]. The development of third-generation RAF inhibitors, including Pan-RAF kinase inhibitors, aims to overcome paradoxical MAPK pathway activation seen with second-generation therapies [111,112], although LY3009120 has shown disappointing clinical results [113]. Belvarafenib, however, demonstrated tolerability and anti-tumor activity in a phase I study with advanced solid tumors with RAS or RAF mutations, and its combination with MEK inhibitor cobimetinib is under further investigation [114].

### 3.3. Thyroid Cancer

The BRAF V600E mutation plays a central role in modulating therapeutic response in thyroid cancers, notably by activating the MAPK pathway, which suppresses key proteins involved in iodine uptake, such as the sodium–iodide symporter and the TSH receptor. This mechanism underlies resistance to radioactive iodine therapy in many differentiated thyroid cancers. However, the phase II MERAIODE trial demonstrated that dual MAPK inhibition with dabrafenib and trametinib could restore RAI sensitivity in 95% of patients, with a 38% response rate at 6 months among those harboring BRAF V600E or RAS mutations [115,116]. Beyond differentiated thyroid cancer, this targeted combination has also shown clinical benefit in anaplastic thyroid carcinoma. In the phase II ROAR basket trial, 16 patients with locally advanced or metastatic BRAF V600E-mutated ATC were treated with the same regimen, leading to an objective response rate of 56% and a median overall survival of 14.5 months [19]. These findings supported the FDA approval of dabrafenib plus trametinib for BRAF-mutant ATC in 2018, reinforcing its role across distinct thyroid cancer subtypes [117].

Lastly, the BRAF inhibitor vemurafenib showed effectiveness in BRAFV600E-mutated PTC in a phase II study [118]. Participants were stratified into two cohorts based on prior exposure to VEGFR multi-kinase inhibitors: cohort 1 included treatment-naive individuals, while cohort 2 comprised those previously treated. Cohort 2 showed a lower ORR of 27.3% compared to 38% in cohort 1.

### 3.4. Metastatic Colorectal Cancer

Roughly 10% of metastatic colorectal cancers (mCRCs) harbor the BRAF V600E mutation, a molecular alteration strongly associated with resistance to conventional chemotherapy and a generally poor prognosis [119]. In contrast, tumors with non-V600 BRAF mutations often behave differently, both biologically and clinically, translating into more favorable outcomes [120]. These non-V600 variants typically show reduced kinase activity or rely on upstream RAS signaling to activate the MAPK cascade, leading to less aggressive tumor behavior. Clinically, such tumors are more commonly located on the left side of the colon and tend to exhibit better histological differentiation than their V600E-mutated counterparts [121]. The BEACON phase III trial [72] tested the combination of encorafenib, a targeted BRAF inhibitor, with cetuximab, an EGFR-targeting monoclonal antibody. This regimen extended median overall survival to 8.4 months, compared to 5.4 months with standard chemotherapy, a clinically meaningful 3-month benefit. A third study arm assessed a triplet therapy that included the MEK inhibitor binimetinib. While survival increased slightly to 9.0 months, the added benefit was offset by a higher burden of toxicity, notably retinal effects and elevated CPK levels, well-documented side effects of MEK inhibition. Based on these findings, the FDA approved the encorafenib–cetuximab combination in 2020 for patients with previously treated BRAF V600E-mutated mCRC, marking a shift toward more personalized treatment strategies in this subgroup [122].

The efficacy of combining encorafenib and cetuximab with chemotherapy as a first-line treatment for mCRC with BRAFV600E mutations is currently under review in the phase III BREAKWATER trial (NCT04607421).

Thyroid cancers were excluded from the toxicity table, as chemotherapy is not standard in this setting. Most cases are treated with surgery, radioactive iodine, or targeted therapies.

## 4. Agnostic Approach to BRAF Inhibition: Selected Examples of Rare Tumors

In June 2022, the FDA issued an accelerated approval for the use of dabrafenib combined with trametinib in treating both adults and children aged six and older with solid tumors that are either inoperable or metastatic and possess the BRAFV600E mutation, provided that these tumors have worsened after previous treatments and no other satisfactory treatment options are available [123]. This authorization was supported by significant treatment responses noted in various rarer solid tumors with the BRAFV600E mutation, including but not limited to certain rarer cancers of the bile duct, central nervous system, gynecological tumors, and digestive tract.

**Biliary tract tumors**: BRAF V600E mutations are identified in about 5–7% of biliary tract cancers (BTCs), particularly more frequent in intrahepatic BTCs [124]. Patients with intrahepatic BTC harboring BRAF V600E mutations often present with advanced tumor stages at surgery, increased lymph node involvement, and generally poorer outcomes compared to patients without these mutations [125]. The phase II ROAR trial results from 2023 indicated that dabrafenib and trametinib treatment led to a 53% ORR, a DOR of 8.9 months, and a median PFS of 9 months in the BRAF V600E-mutated BTC cohort [19]. The phase II NCI MATCH EAY131-H trial, which included four patients with BRAF V600E-mutated intrahepatic BTC, showed a 38% ORR and a median PFS of 11.4 months across the board, with 75% of BTC patients achieving partial responses (PRs) [126] (Figure 2).

**Central nervous system tumors**: In central nervous system (CNS) tumors, BRAF mutations occur in about 7% of cases. They are notably prevalent in various subtypes, such as 60% of pleomorphic xanthoastrocytomas, 10–12% of anaplastic xanthoastrocytomas, 80–95% of benign papillary craniopharyngioma, 38% of astroblastoma, 20–70% of gangliogliomas, 10% of pilocytic astrocytoma, and 1–2% of adult glioblastomas (GBMs). Class I BRAF mutations account for 44–66% of all BRAF mutations in gliomas, with class II and III mutations making up 10–24% and 4–10%, respectively [127]. GBMs with BRAF mutations typically exhibit distinct characteristics, including younger patient age and longer survival rates, compared to other GBM cases [128]. The BRAF V600E mutation is more common in IDH-wild-type tumors than in IDH-mutant tumors, now classified as astrocytomas [127]. However, the prognostic impact of BRAF V600E mutations in gliomas remains uncertain. BRAF/MEK inhibitors have limited intracranial efficacy and are theoretically considered unable to effectively cross the blood–brain barrier [129], yet clinical data have demonstrated efficacy in melanoma brain metastases [91]. It is therefore reasonable to hypothesize that BRAF-mutated brain tumors may respond to these agents. Initially, evidence of efficacy was limited to case reports [130] and retrospective analyses [131]. Specifically, in a retrospective study of six patients treated with BRAF/MEK inhibitors after progression on standard therapy, three patients (50%) achieved partial responses and one achieved stable disease, with a median progression-free survival of 6 months. Although limited by sample size, there was a non-statistically significant trend toward improved overall survival in patients receiving BRAF/MEK inhibitors (median OS, 35.6 months), compared to those who did not (median OS, 17.0 months). The VE-BASKET trial assessed vemurafenib in various non-melanoma BRAF V600E mutant tumors, including gliomas, showing a 25% ORR with a median PFS of 5.5 months and a median OS of 28.2 months in the overall population [132]. The ROAR trial also evaluated the dabrafenib–trametinib combination in BRAF V600E-mutated rare tumors, including high-grade and low-grade gliomas, showing a 54% ORR in low-grade glioma (LGG) and a 33% ORR in high-grade glioma (HGG), suggesting superior efficacy over vemurafenib alone [19] (Figure 2).

**Gynecologic tumors**: In gynecological cancers, low-grade serous ovarian carcinoma (LGSOC) represents 5–10% of all epithelial ovarian cancers, characterized by its slow progression, low chemotherapy response rates, and high prevalence of MAPK pathway alterations [133]. BRAF mutations in LGSOC vary across studies but are found in approximately 2–16% of cases [134]. The AACR GENIE cohort identified BRAF mutations in 9.5% of LGSOC cases [135]. The phase III MILO/ENGOT-ov11 trial of binimetinib in LGSOC was discontinued early after interim analysis showed no benefit in PFS, with an ORR of 16% and median PFS of 9.1 months in the binimetinib group versus 10.6 months in the control chemotherapy group [136]. However, a molecular analysis indicated that binimetinib had a higher ORR in patients with MAPK pathway alterations (41%) compared to those without (13%) [137], suggesting potential benefits for some patients. The NCI MATCH EAY131-H trial, including LGSOC patients, showed clinical benefits from dabrafenib and trametinib therapy, with PR in five patients and stable disease in one [126]. Lastly, trametinib monotherapy in recurrent LGSOC in the GOG281/LOGS trial showed improved PFS of 13.0 months compared to 7.2 months in the standard-care group, with a 26% ORR in the trametinib group versus 6% in the standard care group, making it a viable treatment option for LGSOC regardless of BRAF mutation status [138].

**Gastrointestinal stromal tumors**: BRAF V600E mutations are also found in 0.6 to 3.9% of gastrointestinal stromal tumors (GISTs), acting as tumor-agnostic markers predictive of response to BRAF inhibitors, similar to NTRK fusions [139].

## 5. Mechanisms of Resistance

### 5.1. Intrinsic Resistance

Mutationally activated kinases are key therapeutic targets in cancer, but resistance often limits the efficacy of their inhibitors. A common mechanism involves the activation of alternative survival pathways via RTKs, which converge on MAPK and PI3K signaling. RTK ligands, produced by tumor cells, stroma, or systemically, can bypass kinase inhibition and restore downstream signaling. BRAF-mutant melanoma cells can be rescued from BRAF inhibitor-induced growth arrest by various RTK ligands. Hepatocyte growth factor HGF reactivates both MAPK and PI3K, while IGF and NRG1 activate PI3K, and EGF or FGF reactivates MAPK. Co-targeting these secondary pathways can reverse resistance, as shown with PI3K inhibition in HGF-driven models. IGF1R is a relevant RTK in this context, and its inhibition suppresses AKT and ERK signaling even in BRAF-mutant cells, regardless of NRAS or phosphatase and TENsin homolog (PTEN) status, highlighting alternative routes for MAPK reactivation. Tumor heterogeneity further contributes to resistance. Pre-existing subclones responsive to specific RTK ligands may expand under treatment pressure. In BRAF-mutant melanoma, heterogeneous MET expression allows for HGF-driven resistance. Similar adaptive shifts to alternative RTKs have been observed in EGFR-mutant NSCLC and glioblastoma. These findings emphasize the redundancy and flexibility of RTK signaling and support combination strategies targeting both primary and compensatory pathways [140,141].

Intrinsic resistance in BRAF-mutant tumors can also be caused by alterations in cell cycle regulation, particularly involving cyclin-dependent kinase 4 (CDK4) and cyclin D1 (CCND1). Mutations in BRAF typically drive uncontrolled proliferation by activating the MAPK pathway, leading to increased expression of CCND1, which regulates CDK4 activity, thus facilitating cell cycle entry. Studies of melanoma cells with BRAF V600E mutations showed that CDK4 mutations alone did not significantly affect response to BRAF inhibitors. However, resistance significantly increased when CDK4 mutations occurred alongside CCND1 amplification [142].

Alterations in the MAPK pathway can also lead to resistance in BRAF-driven tumors [143]. Elevated copy numbers of MAP3K8 (COT) have been linked to resistance through MAPK pathway reactivation. In another study, the loss of NF1, a negative regulator of RAS/MAPK signaling, was shown to contribute to sustained MAPK pathway activation and resistance to RAF and MEK inhibitors [144]. RAC1 gain-of-function alterations have also been identified as intrinsic resistance mechanisms, with pre-existing high-activity RAC1 cells being selected during drug resistance [145].

Resistance to MAPK inhibitors can also be mediated by the Hippo pathway effector Yes-associated protein (YAP), a transcriptional co-activator. Genetic screens have identified YAP as a mediator of resistance to BRAF-inhibitor treatment. Elevated YAP1 expression is associated with this resistance in preclinical models and has been correlated with poor survival in patient cohorts with melanoma and NSCLC treated with BRAF inhibitors. Inhibiting YAP genetically can reverse resistance in these models. YAP’s role in resistance against BRAF-targeted therapy may be both an intrinsic and an adaptive response [146].

Androgen receptor (AR) expression has emerged as a potential resistance mechanism to BRAF/MEK inhibitors, with preclinical and clinical data indicating a role in gender-related treatment differences. In vivo, male mice exhibited reduced responses to BRAF/MEK-targeted therapy compared to females, an effect linked to elevated AR signaling. Clinical data support this observation, suggesting that AR activity, and possibly patient sex, may impact outcomes in BRAF-mutant melanoma. Mechanistically, AR sustains MAPK signaling and promotes proliferation via upregulation of cyclin D1 and other cell cycle regulators, counteracting the cytostatic effects of BRAF/MEK inhibition. It also fosters transcriptional reprogramming and cellular plasticity associated with adaptive resistance. Preclinical studies show that AR blockade can reverse these effects, enhancing apoptosis and improving response to BRAF/MEK inhibitors. These findings support exploring combination strategies with AR and MAPK pathway inhibitors in AR-expressing BRAF-mutant melanomas [123,147].

### 5.2. Acquired Resistance

While intrinsic resistance is relatively rare, **acquired resistance** following treatment with BRAF inhibitors is almost inevitable. A multitude of mechanisms contribute to this phenomenon, with reactivation of the MAPK pathway, often referred to as “MAPK reactivation” or “MAPK addiction”, being the most prominent [123,148]. This can result from secondary mutations in KRAS, NRAS, MAP2K1, and MAP2K2, or from amplifications in BRAF itself. Additionally, splice variants of BRAFV600E that promote RAF dimerization can bypass inhibitor effects. Alterations in the PI3K/PTEN pathway, such as mutations in PIK3CA, PTEN, AKT1, PIK3R1, PIK3R2, and AKT3, can drive resistance independently of MAPK reactivation [123].

The loss of the tumor suppressor PTEN has also been associated with a poorer prognosis following BRAF-inhibitor treatment. In a study by Nathanson et al. [149], melanomas with baseline loss or mutations of PTEN showed a trend toward shorter median progression-free survival compared to those with normal PTEN expression. PTEN normally acts as a negative regulator of the PI3K/AKT pathway, which promotes cell survival and proliferation. Its loss leads to pathway hyperactivation, allowing melanoma cells to bypass MAPK inhibition, even under BRAF or MEK-targeted therapy. PTEN loss has also been linked to reduced progression-free and overall survival [150,151], and it may contribute to an immunosuppressive tumor microenvironment [152].

Similarly, CDKN2A mutations or deletions are associated with adverse outcomes in BRAF-mutant melanoma. CDKN2A encodes the tumor suppressor p16INK4a, which regulates the G1/S cell cycle checkpoint via inhibition of CDK4/6. Loss of CDKN2A results in uncontrolled cell cycle progression, correlating with more aggressive disease, earlier metastasis, and therapy resistance. These alterations are common in BRAF-mutant melanoma and are associated with shorter survival. Importantly, they also support the rationale for evaluating CDK4/6 inhibitors in combination with BRAF-targeted therapies [153].

In non-small-cell lung cancer, acquired resistance to BRAF/MEK inhibitors often involves mutations in MEK1, PTEN, NRAS, and KRAS [154]. In melanoma, a significant proportion of resistant tumors progress without detectable genetic alterations. In these cases, resistance is likely driven by epigenetic and transcriptomic reprogramming, including DNA methylation, histone modifications, and miRNA-mediated gene regulation [147].

Further studies have revealed that resistance is not only molecular but phenotypic. Long-term BRAF inhibition induces divergent cellular states; some resistant cells remain proliferative, while others become slow-cycling and sensitive to ferroptosis [155]. This opens up the possibility of targeting non-MAPK-related resistance mechanisms. For example, antiretroviral agents like doravirine can restore apoptosis in resistant melanoma cells by upregulating tumor suppressors such as p16INK4a and p27Kip1 [155].

Other resistance pathways involve compensatory signaling networks. Activation of the EGFR–YAP1–TEAD2 axis promotes stromal interaction molecule 1 (STIM1) upregulation and calcium signaling in vemurafenib-resistant cells [156], while ERK5 signaling enhances survival in dabrafenib-resistant cells and contributes to MEK inhibitor resistance [157].

Resistance is also linked to enhanced invasiveness. The cytoskeletal effector MARCKS, upregulated in BRAF inhibitors-resistant melanoma, is critical for cell migration and metastasis. Its activity is driven by protein kinase C (PKC) and RhoA, and dual inhibition of these proteins reduces MARCKS activity and invasive behavior [158].

Interestingly, in a retrospective analysis of 113 cutaneous melanoma samples assessed by NGS, 86.8% of BRAFV600-mutant tumors harbored at least one additional mutation. The most frequent co-mutation involved the Telomerase reverse transcriptase (TERT) promoter (73.6%). CDKN2A and PTEN mutations were also observed in 17.0% and 15.1% of cases, respectively. Less common alterations included mutations in NRAS, MAP2K1, RAC1, IDH1, PIK3CA, STK11, ERBB4, GNAS, and GNA11. In comparison with BRAF wild-type cases, NRAS mutations were significantly less frequent in BRAFV600 samples (3.8% vs. 44.4%). Some mutations were exclusive to either BRAFWT (e.g., KIT, CTNNB1, and MET) or BRAFV600 samples (e.g., MAP2K1, STK11, PIK3CA, GNAS, and GNA11) [159].

## 6. Conclusions

Major efforts are underway to improve the effectiveness of targeted therapies, particularly BRAF inhibitors. Improving drug properties such as blood–brain barrier permeability is critical for better management of patients with brain metastases [160,161]. Another key priority is the development of next-generation inhibitors capable of targeting a broader range of BRAF alterations, including non-V600 mutations [162].

The emergence of tissue-agnostic strategies, supported by basket trials such as the ROAR study, has offered valuable insights into how tumors across different organs respond to BRAF-targeted therapies. These trials have laid the groundwork for mutation-driven treatment paradigms. However, they also highlight the limitations of applying a uniform approach across tumor types. The biological behavior of a BRAF-mutant tumor is profoundly influenced by its tissue of origin, co-occurring mutations, and microenvironmental context [163]. For instance, while BRAF and MEK inhibitors show synergistic potential with immunotherapy in melanoma, this combination has limited efficacy in lung cancer. In colorectal cancer, clinical benefit requires the addition of EGFR inhibitors to overcome pathway reactivation. Such differences highlight the necessity of tailoring therapeutic strategies to the specific molecular and cellular context of each cancer type.

Toxicity remains a major challenge, particularly in the development of combination regimens involving BRAF/MEK inhibitors and immunotherapy. Managing these adverse effects while preserving therapeutic benefit is essential for expanding the use of these combinations. Thus, while basket trials have advanced the field of precision oncology, the future lies in developing organ-specific approaches that integrate tumor biology with rational combination strategies (Table 2), all while maintaining a careful balance between efficacy and tolerability.

## Figures and Tables

**Table 1:** Comparative toxicities between chemotherapy and BRAF/MEK inhibitors in the most common cancers.

Cancer Type	Treatment Regimen	Main AEs, Any Grade	Source
**Metastatic melanoma**	Dabrafenib + trametinib	Pyrexia (52%), alanine aminotransferase increase (10%), fatigue (27%), rash (24%), cutaneous squamous cell carcinoma (3%), decreased ejection fraction (4%)	[69]
	Chemotherapy (dacarbazine)	Nausea (14%), vomiting (5%), fatigue (5%), diarrhea (15%), hematological toxicities (>15%)	[70]
**Metastatic non-small-cell lung cancer (NSCLC)**	Dabrafenib + trametinib	Pyrexia (46%), nausea (40%), vomiting (35%), asthenia (28%), rash (19%), squamous cell carcinoma of skin (4%)	[18]
	Standard second-line chemotherapy (paclitaxel, bevacizumab)	Hematological toxicity (73,4%), neuropathy (49,5%), alopecia (29,4%), vomiting (13,8%)	[71]
**Metastatic colorectal cancer**	Encorafenib + binimetinib + cetuximab	Diarrhea (62%), acneiform dermatitis (49%), nausea (45%), vomiting (38%), pyrexia (20%)	[72]
	Standard chemotherapy (FOLFIRI–cetuximab)	Diarrhea (48%), acneiform dermatitis (39%), nausea (41%), vomiting (29%), pyrexia (14%)	[72]

AEs: adverse events.

**Table 2:** Ongoing BRAF-targeted therapy: A summary of ongoing clinical trials.

Tumor Type	Patient Population	Intervention	Phase	Trial Designation	Status
Melanoma	Patients with resectable stage IIIB-C BRAF V600 mutation-positive melanoma	Dabrafenib + trametinib	II	NCT01972347	NR
	Metastatic or unresectable melanoma carrying a BRAF V600 mutation and having relapsed on a BRAF/MEK inhibitor therapy	Dabrafenib + trametinib	I	NCT04903119	R
	Advanced melanoma patients with BRAF V600E/K mutation	HL-085 + vemurafenib	II	NCT05263453	R
	Unresectable BRAF-mutated stage III/IV melanoma	XL888 + vemurafenib + cobimetinib	II	NCT02721459	NR
	Metastatic or unresectable melanoma carrying a BRAF V600 mutation and having relapsed on a BRAF/MEK inhibitor therapy	Nilotinib + dabrafenib/trametinib or encorafenib/binimetinib	I	NCT04903119	R
	Chinese patients with stage III BRAF V600 mutation positive melanoma after complete resection	Dabrafenib + trametinib	II	NCT04666272	R
	High-risk patients with stage II melanoma with BRAF mutations (COLUMBUS-AD)	Encorafenib + binimetinib	III	NCT05270044	NR
	Treatment-naive patients with advanced/metastatic melanoma with BRAF alterations (STEABOARD)	Encorafenib + binimetinib + pembrolizumab	III	NCT04657991	NR
	BRAF mutant metastatic melanoma (CELEBRATE)	Encorafenib + binimetinib + palbociclib	I/II	NCT04720768	R
	Patients with pretreated advanced melanoma (RegoMel)	Regorafenib	II	NCT05370807	R
	BRAFV600-mutated melanoma with CNS metastasis	E6201 + dabrafenib	I	NCT05388877	NR
mCRC	First-line metastatic colorectal cancer with BRAFV600E mutation	Encorafenib + cetuximab + mFOLFOX6	III	NCT04607421	NR
	Metastatic colorectal cancer (mCRC) with BRAF V600E mutation after first-line treatment	HLX208 (BRAF V600E Inhibitor) + cetuximab	II	NCT04984369	NR
	First-line treatment for RAS/BRAF wild-type advanced colorectal cancer	Sintilimab + cetuximab + chemotherapy	I/II	NCT06776757	R
	Previously treated MSS mCRC with BRAFV600E mutation	Encorafenib + cetuximab + nivolumab	II	NCT05308446	R
	Previously untreated metastatic CRC (BREAKWATER)	Encorafenib + cetuximab +/− chemotherapy	III	NCT04607421	NR
	Previously untreated BRAFV600E mutant, MSI high/DMMR metastatic CRC (SEAMARK)	Encorafenib + cetuximab + pembrolizumab vs. pembrolizumab alone	II	NCT05217446	NR
	BRAF V600E-mutated MSS initially resectable/potentially resectable advanced colorectal cancer	Cetuximab + encorafenib + binimetinib	II	NCT06207656	R
Thyroid	High-risk BRAFV600E-mutant differentiated thyroid carcinoma (pre-radioiodine therapy)	Vemurafenib + cobimetinib	II	NCT06440850	R
	Radioiodine-refractory BRAFV600E-mutant differentiated thyroid cancer (post-VEGFR TKI)	Dabrafenib + trametinib vs. cabozantinib	III	NCT06475989	R
	Previously treated patients with locally advanced/metastatic, RAI-refractory BRAFV600E-mutated differentiated thyroid cancer	Dabrafenib + trametinib	III	NCT04940052	NR
	Patients with metastatic radioiodine refractory BRAFV600 mutant thyroid cancer	Encorafenib + binimetinib +/− nivolumab	II	NCT04061980	NR
NSCLC	Metastatic NSCLC with BRAF V600E mutation (no prior BRAF/MEK inhibitors)	Encorafenib + binimetinib	II	NCT03915951	NR
	Untreated metastatic NSCLC with BRAFV600E mutation	Encorafenib + binimetinib	II	NCT04526782	NR
	Patients with NSCLC, solid tumors, melanoma, high-grade gliomas	Dabrafenib + trametinib	IV	NCT03340506	R
	Advanced NSCLC	Binimetinib + pembrolizumab	I	NCT03991819	R
Other solid tumors	Advanced solid tumors with non-V600E BRAF mutations (e.g., class II/III alterations)	Encorafenib + binimetinib	II	NCT03839342	NR
	Relapsed or progressive pediatric low-grade glioma with BRAF alterations (V600E or BRAF fusions)	Tovorafenib	II	NCT04775485	R
	Advanced solid tumors with oncogenic BRAF (class I/II/III) or RAS/MAPK pathway mutations	BDTX-4933 (pan-RAF/RAS inhibitor)	I	NCT05786924	NR
	adults with BRAF/NRAS-mutated advanced or metastatic solid tumors	KIN-2787	I	NCT04913285	R
	Advanced solid tumors with BRAFV600 mutations (monotherapy dose escalation; combination with trametinib in expansion)	CFT1946 (BRAF V600 degrader) ± trametinib	I/II	NCT05668585	R
	Recurrent/progressive low-grade ovarian or peritoneal cavity cancer	Trametinib vs. standard of care	II/III	NCT02101788	NR
	Patients with BRAF and other RAS/MAPK mutation-positive neoplasms	BDTX-4933	I	NCT05786924	NR
	Advanced solid tumors with BRAF, KRAS, and/or NRAS mutations	VS6766 +/− everolimus	I	NCT02407509	R
	Advanced/metastatic malignancies harboring RAS or RAF oncogenic mutations	IMM-6-415	I/II	NCT06208124	R
	Advanced/recurrent low-grade glioma or pancreatic cancer with BRAF fusion/rearrangement	Binimetinib	II	NCT06159478	R

AJCC, American Joint Committee on Cancer; BRAF V600E, a common activating mutation in the BRAF gene; CNS, central nervous system; CRC, colorectal cancer; DMMR, deficient mismatch repair; MSS, microsatellite stable; MSI-H, microsatellite instability-high; mCRC, metastatic colorectal cancer; NSCLC, non-small-cell lung cancer; ORR, objective response rate; OS, overall survival; PFS, progression-free survival; RAI, radioactive iodine; RAS, rat sarcoma virus protein; RAF, rapidly accelerated fibrosarcoma kinase (including ARAF, BRAF, and CRAF); MAPK, mitogen-activated protein kinase; MEK, MAPK/ERK kinase (also known as MAP2K1/2); TKI, tyrosine kinase inhibitor; VEGFR, vascular endothelial growth factor receptor; WT, wild-type; R, recruiting; NR, active, non-recruiting.
